# Supplementary material for: A multivariate statistical evaluation of actual use of electronic health record systems implementations in Kenya
Source: PLoS One. 2021 Sep 7;16(9):e0256799. doi: 10.1371/journal.pone.0256799 (PMC8423313; doi:10.1371/journal.pone.0256799)

S1 Appendix. Distribution of KeEMRs implementations as of June 2020

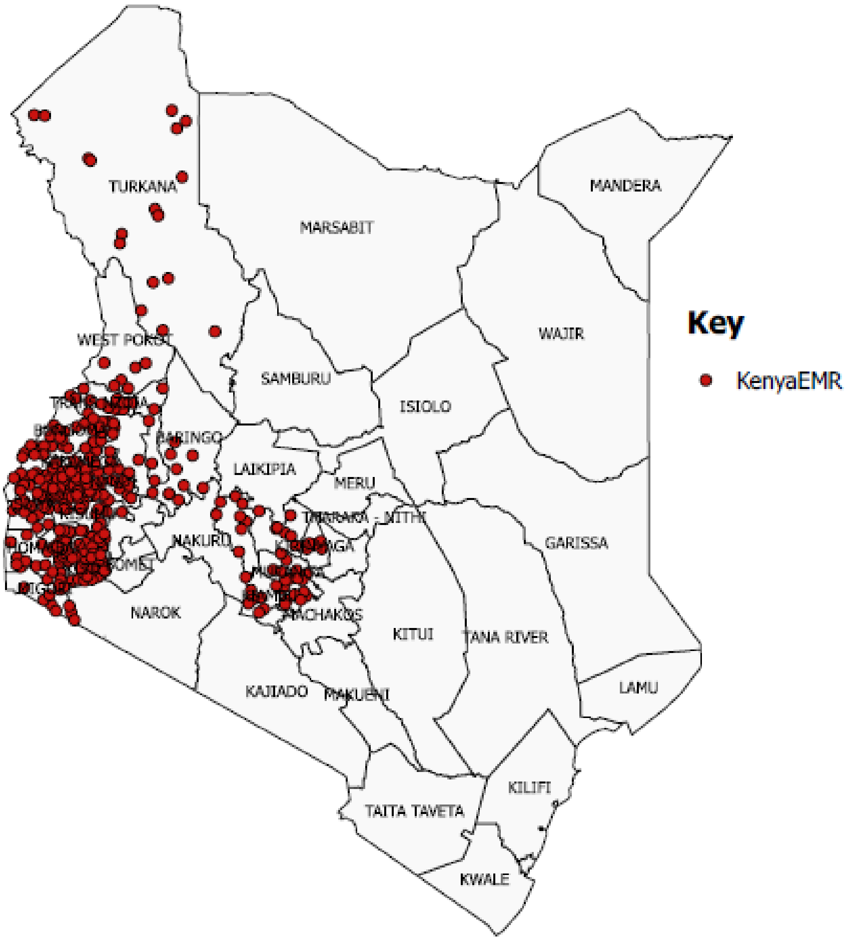

Reprint from https://dwh.nascop.org/#/ under a CC BY license, with permission from The Palladium Group- KeHMIS II Project, original copyright 2020

The chart shows frequencies and percentages of the five types of EHRs implementations in Kenya by June 2020, showing that 31% are KenyaEMR sites.

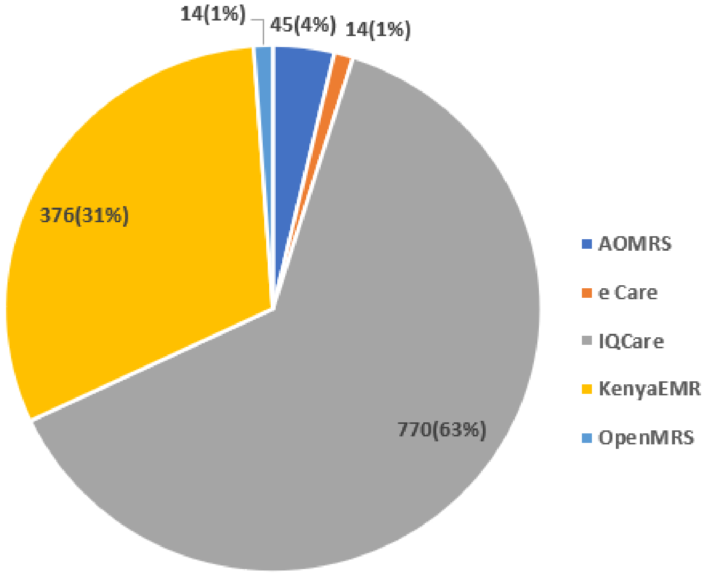

Supplement: S1 Appendix — (PDF) [file pone.0256799.s001.pdf]
