# Supplementary material for: A multivariate statistical evaluation of actual use of electronic health record systems implementations in Kenya
Source: PLoS One. 2021 Sep 7;16(9):e0256799. doi: 10.1371/journal.pone.0256799 (PMC8423313; doi:10.1371/journal.pone.0256799)
Supplement: S2 Appendix — (PDF) [file pone.0256799.s002.pdf]

S2 Appendix. Standard operating procedures for query extraction

Objective: This Standard operating procedure outlines the procedure for extracting queries provided

Context:

*The query has been provided in a zipped folder/directory named Queryscript**.zip**. Extract it to the Desktop in ubuntu machine and use it to complete this exercise.*

Configure KeEMR Data Tool

1. Launch data tool application on the desktop.
2. On the Metadata explorer right click on MySQL to create a new data source connection.

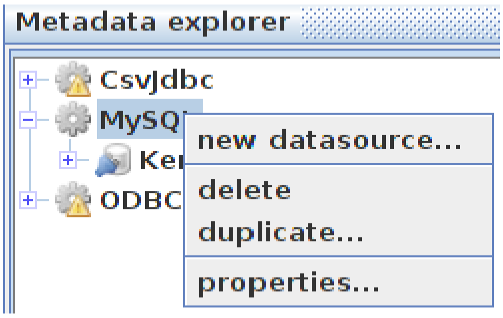

3. Select new datasource and enter the following details :
   a. Data source name: **"OpenMRS"**
   b. URL: **jdbc:mysql://localhost:3306/openmrs**
   c. User name: **root**
   d. Password: **test (Input the mysql password)**
   e. Check "**Remember Password**", "**Auto-connect on start-up**" and "**connect**" check boxes and click "**OK**"

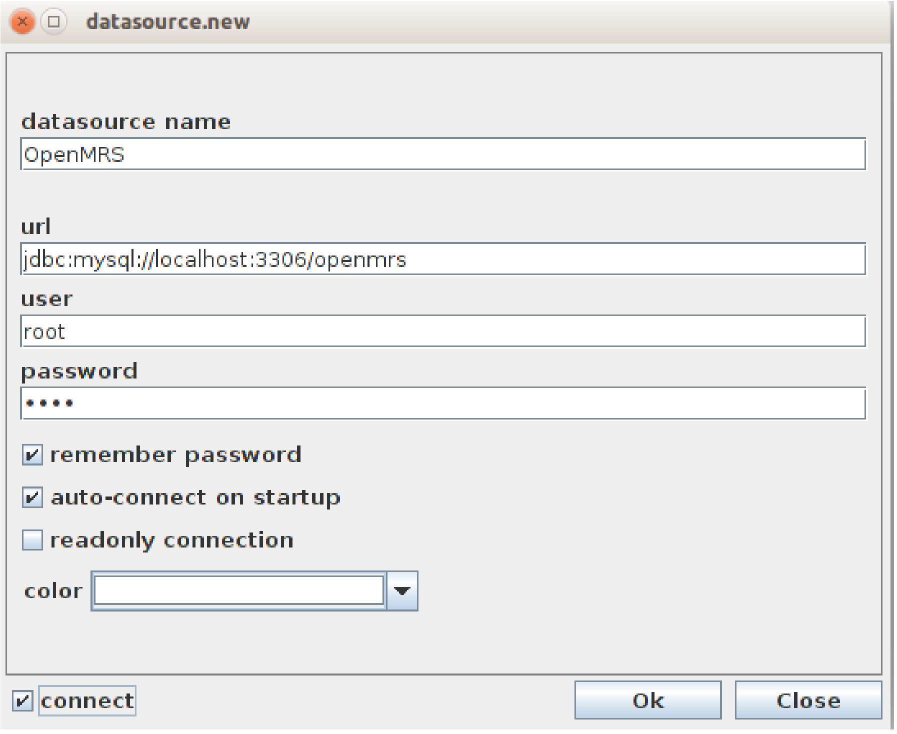

4. Double click on the OpenMRS connection and click on the table to ensure the tables are displayed to the right window as shown below.

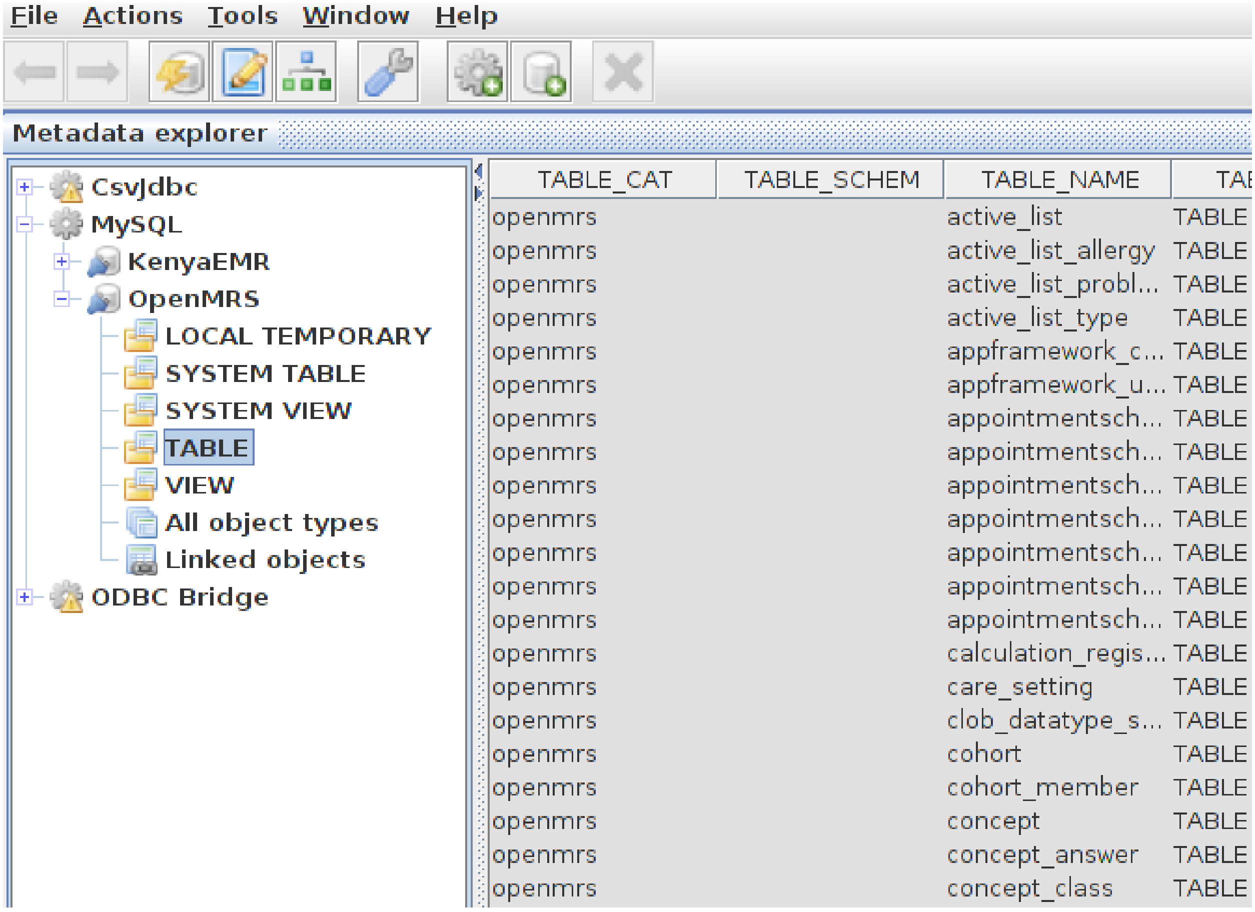

5. Navigate to the Desktop in the terminal by typing Cd Desktop then press enter. Then navigate to the combined -query folder and press enter .

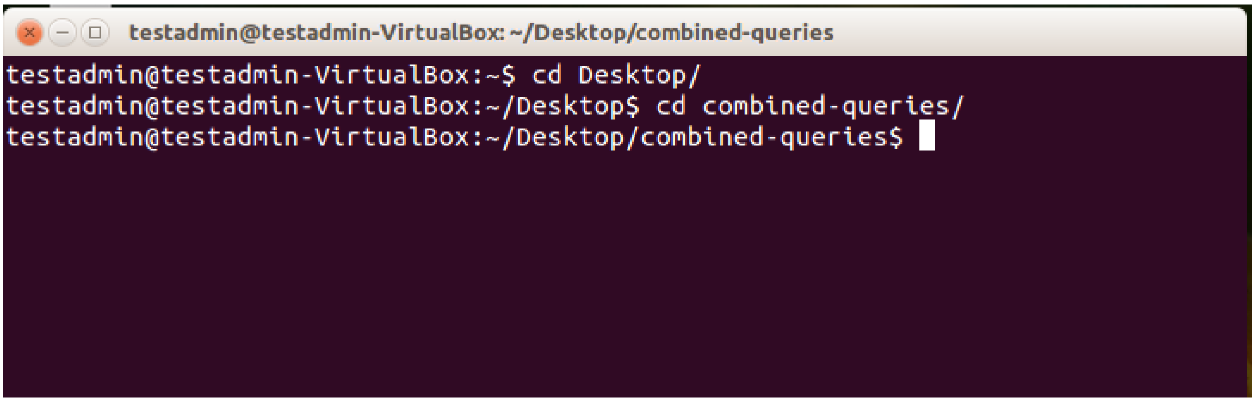

6. Then type the following command and press enter
sudo chmod a+x run_query.sh
Enter password for Admin account

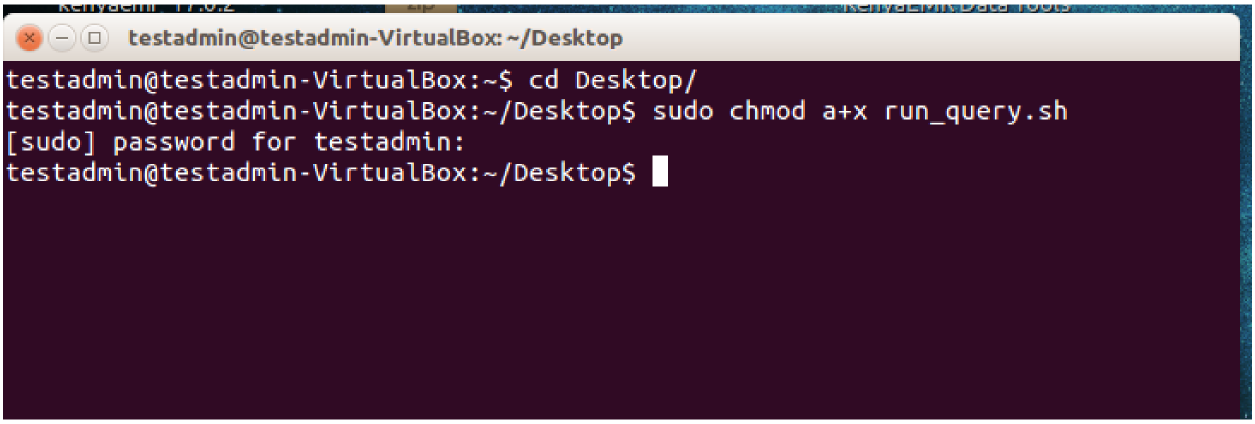

7.Then type sudo ./run_query.sh and press enter

Enter mysql password for root and press enter.(NB:When you input mysql root password it will show on the terminal)

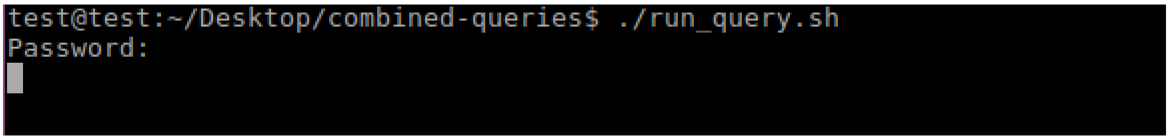

8. Once you have entered the password the query will start executing as shown below.

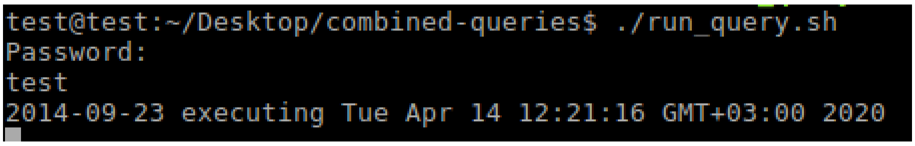

9. Wait for the query to complete execution then input mysql root password once again and press enter as shown below as shown below.(NB:Mysql root password will not show)

```
2014-09-23 executing Tue Apr 14 12:21:16 GMT+03:00 2020
Finished Execution
Enter password:
```

10. After inputting mysql root password, you will be prompted to input the password for the admin account. Input the password then press enter.

```
2014-09-23 executing Tue Apr 14 12:21:16 GMT+03:00 2020
Finished Execution
Enter password:
[sudo] password for test:
```

11. Wait for the queries to finish executing.

```
[illegible]
[sudo] password for test:
tar: Removing leading `/' from member names
/tmp/emr_usage_indicators/cccIdentifierConformance.csv
/tmp/emr_usage_indicators/summaryDataForRDQAItems.csv
/tmp/emr_usage_indicators/numberOfActiveUsersPerRoleMonthAndYearN.csv
/tmp/emr_usage_indicators/numberOfActiveUsersPerRoleMonthAndYearD.csv
/tmp/emr_usage_indicators/numberOfActiveUsersPerMonthAndYearD.csv
/tmp/emr_usage_indicators/numberOfActiveUsersPerMonthAndYearN.csv
/tmp/emr_usage_indicators/reportingRequestsCount.csv
/tmp/emr_usage_indicators/ilMessagesCount.csv
[illegible]
```

12. Navigate to the tmp folder.

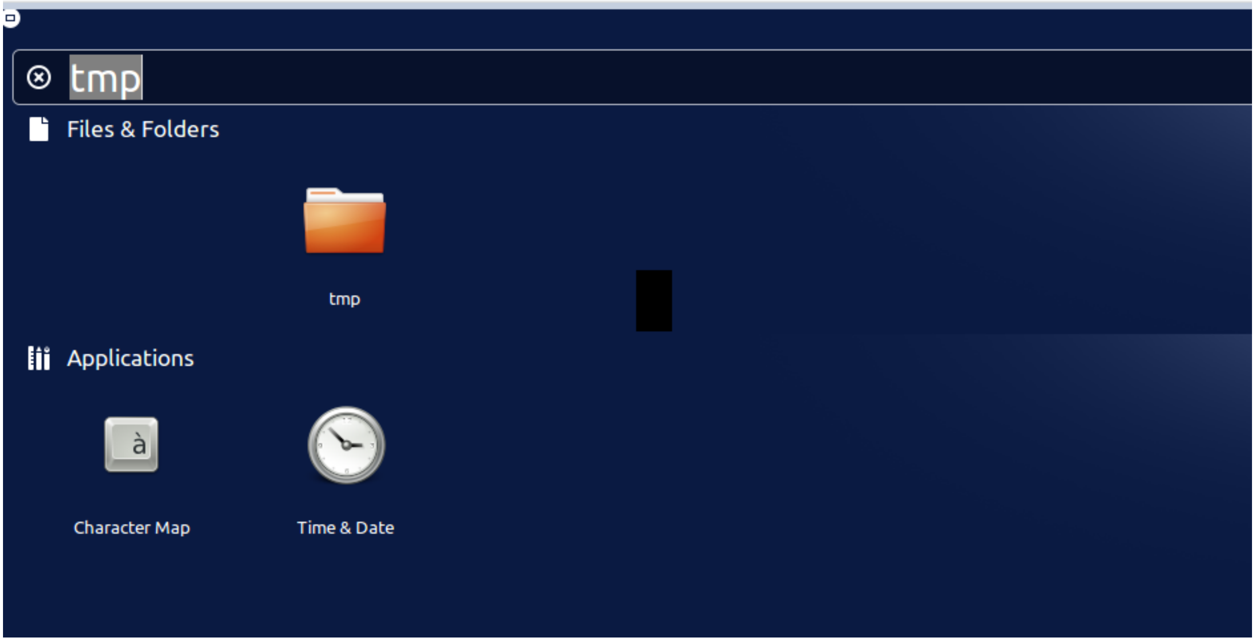

13. Open the tmp folder and get to a folder named **emr_usage_indicators**

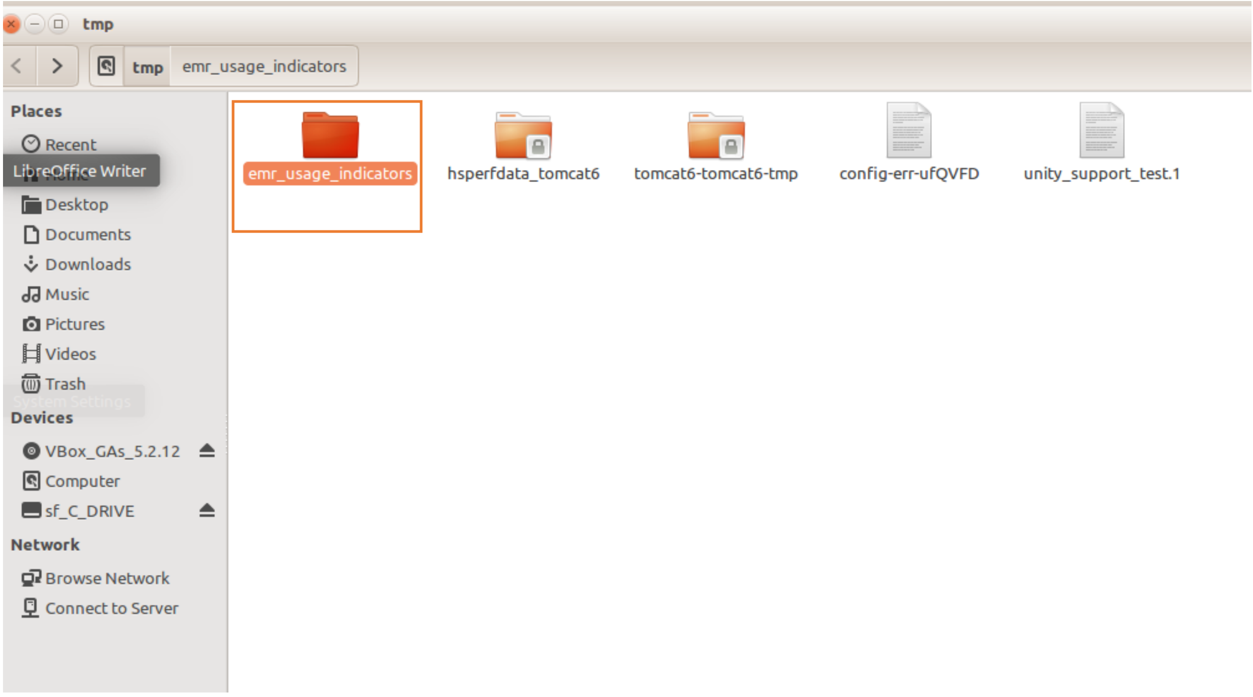

14. On right click you get these options

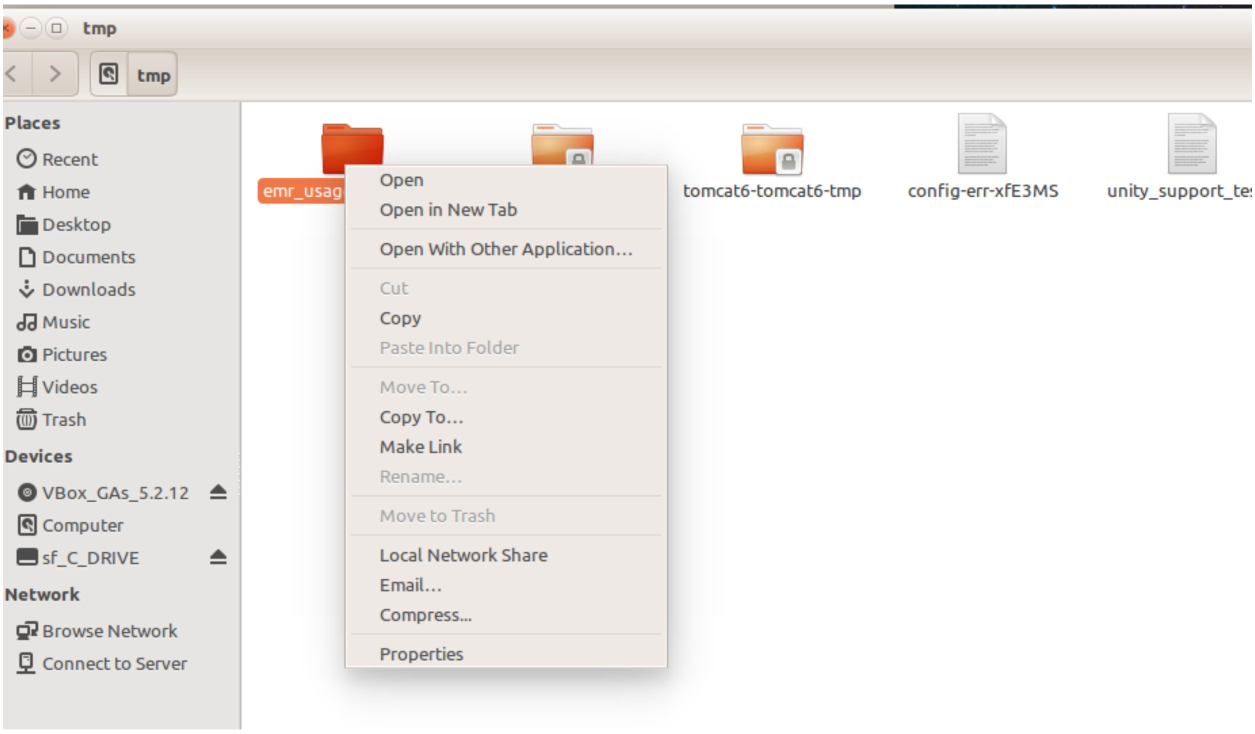

15. Select Compress then click create

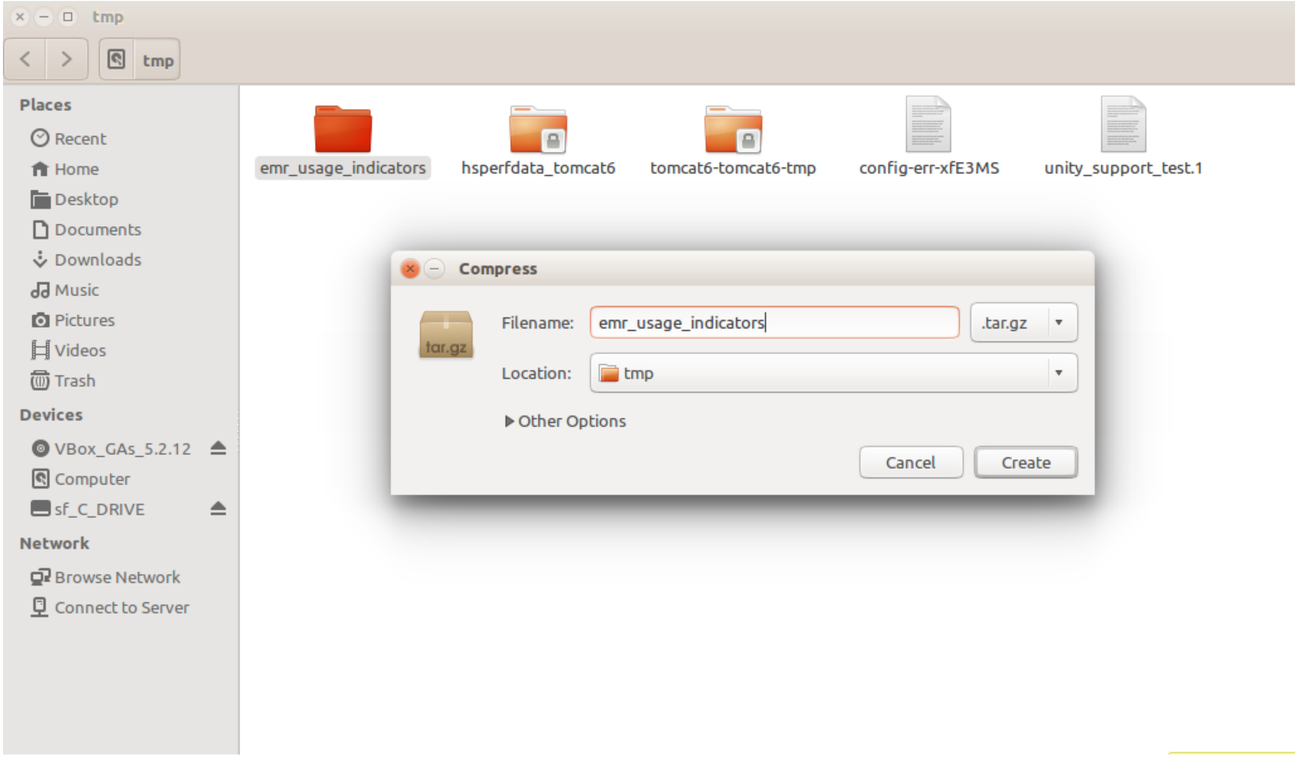
tmp
Places
Recent
Home
Desktop
Documents
Downloads
Music
Pictures
Videos
Trash
Devices
VBox_GAs_5.2.12
Computer
sf_C_DRIVE
Network
Browse Network
Connect to Server
emr_usage_indicators
hsperfdata_tomcat6
tomcat6-tomcat6-tmp
config-err-xfE3MS
unity_support_test.1
Compress
Filename:
emr_usage_indicators
.tar.gz
Location:
tmp
Other Options
Cancel
Create

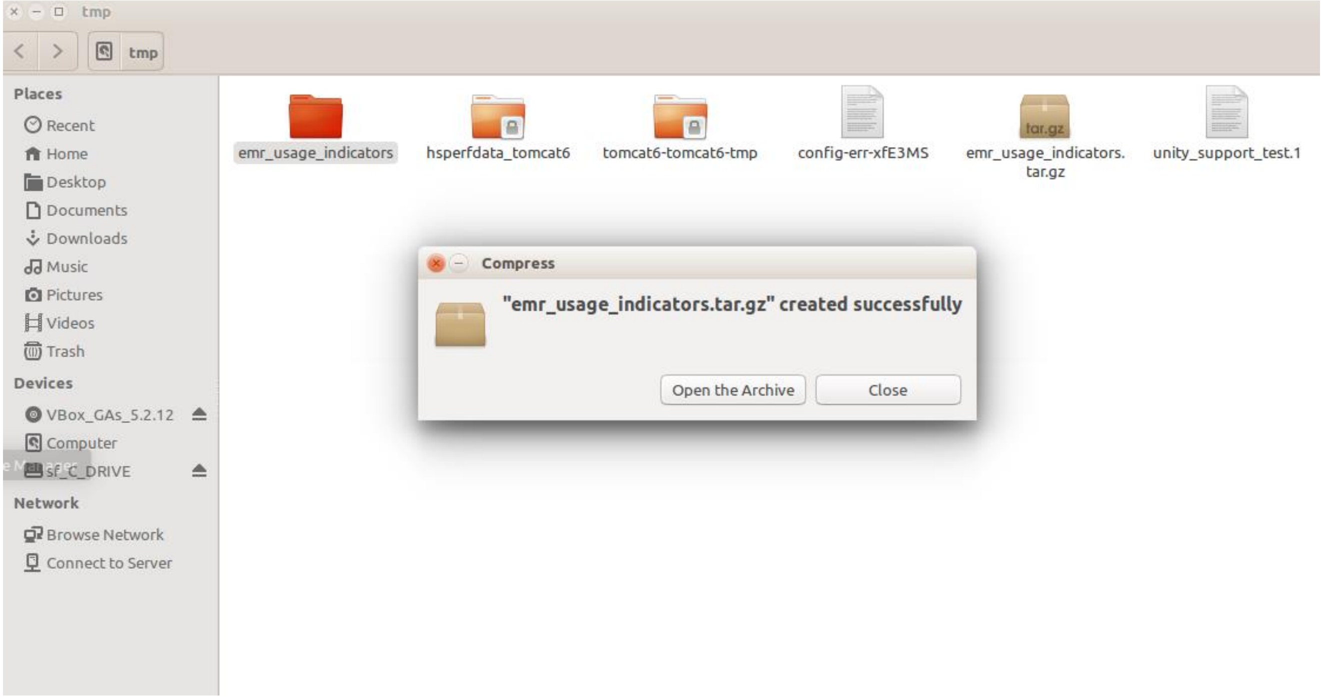
tmp
Places
Recent
Home
Desktop
Documents
Downloads
Music
Pictures
Videos
Trash
Devices
VBox_GAs_5.2.12
Computer
sf_C_DRIVE
Network
Browse Network
Connect to Server
emr_usage_indicators
hsperfdata_tomcat6
tomcat6-tomcat6-tmp
config-err-xfE3MS
emr_usage_indicators.tar.gz
unity_support_test.1
Compress
"emr_usage_indicators.tar.gz" created successfully
Open the Archive
Close

16. Go back to the tmp folder and find the zipped folder [**emr_usage_indicators.tar.gz**]

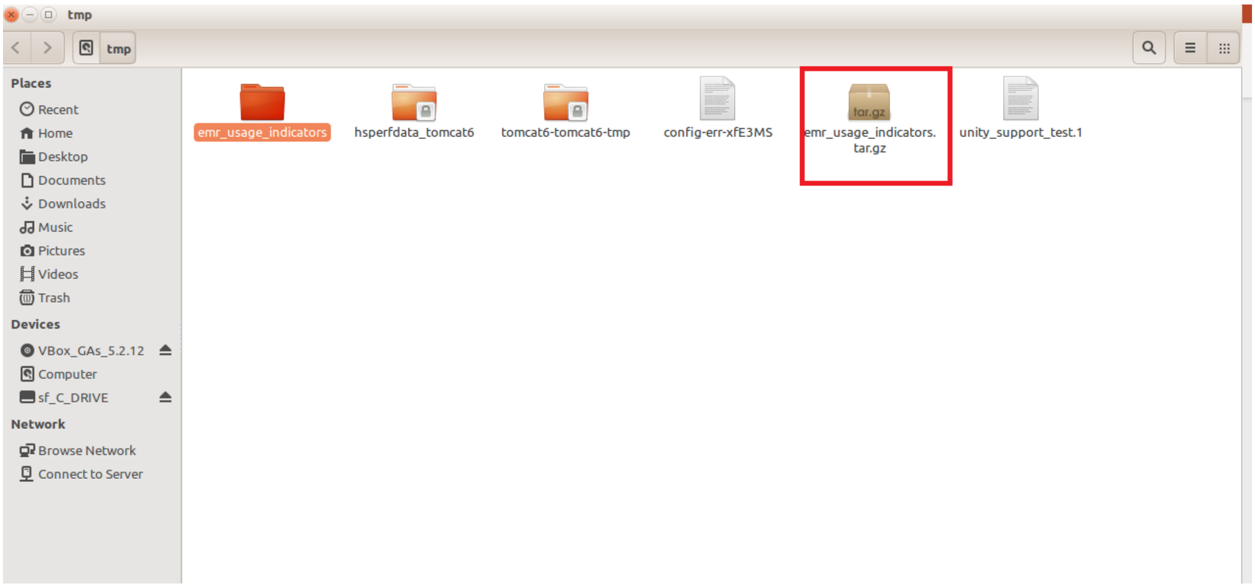

17. Email the zipped folder **emr_usage_indicators.tar.gz** as an attachment to philomenangugi02@gmail.com

Should you have any problem running these queries, please call helpdesk on toll-free number on **0800-722440**

<<END>>
