# Supplementary material for: A multivariate statistical evaluation of actual use of electronic health record systems implementations in Kenya
Source: PLoS One. 2021 Sep 7;16(9):e0256799. doi: 10.1371/journal.pone.0256799 (PMC8423313; doi:10.1371/journal.pone.0256799)
Supplement: S3 Appendix — (PDF) [file pone.0256799.s003.pdf]

S3 Appendix. KeEMRs implementations distribution in the period 2012-2019 across the counties (n=19)

County		Implementation Year					Total
		2012	2013	2014	2018	2019	
A	Count	0	3	6	0	0	9
	%	.0%	33.3%	66.7%	.0%	.0%	100.0%
B	Count	1	5	4	0	0	10
	%	10.0%	50.0%	40.0%	.0%	.0%	100.0%
C	Count	0	4	3	0	0	7
	%	.0%	57.1%	42.9%	.0%	.0%	100.0%
D	Count	0	4	0	0	0	4
	%	.0%	100.0%	.0%	.0%	.0%	100.0%
E	Count	1	8	16	0	0	25
	%	4.0%	32.0%	64.0%	.0%	.0%	100.0%
F	Count	0	5	15	0	0	20
	%	.0%	25.0%	75.0%	.0%	.0%	100.0%
G	Count	0	4	7	0	1	12
	%	.0%	33.3%	58.3%	.0%	8.3%	100.0%
H	Count	0	3	1	0	0	4
	%	.0%	75.0%	25.0%	.0%	.0%	100.0%
I	Count	0	6	10	0	0	16
	%	.0%	37.5%	62.5%	.0%	.0%	100.0%
J	Count	1	4	2	2	1	10
	%	10.0%	40.0%	20.0%	20.0%	10.0%	100.0%
K	Count	0	8	10	0	0	18
	%	.0%	44.4%	55.6%	.0%	.0%	100.0%
L	Count	0	5	5	0	0	10
	%	.0%	50.0%	50.0%	.0%	.0%	100.0%
M	Count	2	5	5	0	0	12
	%	16.7%	41.7%	41.7%	.0%	.0%	100.0%
N	Count	0	7	11	0	0	18
	%	.0%	38.9%	61.1%	.0%	.0%	100.0%
O	Count	0	4	2	0	0	6
	%	.0%	66.7%	33.3%	.0%	.0%	100.0%
P	Count	0	6	3	0	0	9
	%	.0%	66.7%	33.3%	.0%	.0%	100.0%
Q	Count	0	3	4	0	0	7
	%	.0%	42.9%	57.1%	.0%	.0%	100.0%
R	Count	0	5	8	0	0	13
	%	.0%	38.5%	61.5%	.0%	.0%	100.0%
S	Count	0	2	1	0	0	3
	%	.0%	66.7%	33.3%	.0%	.0%	100.0%
Total	Count	5	91	113	2	2	213
	%	2.3%	42.7%	53.1%	.9%	.9%	100.0%
