# Supplementary material for: A multivariate statistical evaluation of actual use of electronic health record systems implementations in Kenya
Source: PLoS One. 2021 Sep 7;16(9):e0256799. doi: 10.1371/journal.pone.0256799 (PMC8423313; doi:10.1371/journal.pone.0256799)
Supplement: S5 Appendix — (PDF) [file pone.0256799.s005.pdf]

S5 Appendix. Interoperability layer (IL) module (data exchange) presence/absence in facilities across the counties

			Data exchange capability (%)		Total
			Absent	Present	
County	A	Count	9	0	9
		% within County	100.0%	.0%	100.0%
	B	Count	10	0	10
		% within County	100.0%	.0%	100.0%
	C	Count	7	0	7
		% within County	100.0%	.0%	100.0%
	D	Count	4	0	4
		% within County	100.0%	.0%	100.0%
	E	Count	21	4	25
		% within County	84.0%	16.0%	100.0%
	F	Count	19	1	20
		% within County	95.0%	5.0%	100.0%
	G	Count	12	0	12
		% within County	100.0%	.0%	100.0%
	H	Count	4	0	4
		% within County	100.0%	.0%	100.0%
	I	Count	16	0	16
		% within County	100.0%	.0%	100.0%
	J	Count	6	4	10
		% within County	60.0%	40.0%	100.0%
	K	Count	16	2	18
		% within County	88.9%	11.1%	100.0%
	L	Count	8	2	10
		% within County	80.0%	20.0%	100.0%
	M	Count	12	0	12
		% within County	100.0%	.0%	100.0%
	N	Count	1	17	18
		% within County	5.6%	94.4%	100.0%
	O	Count	6	0	6
		% within County	100.0%	.0%	100.0%

	P	Count	9	0	9
		% within County	100.0%	.0%	100.0%
	Q	Count	7	0	7
		% within County	100.0%	.0%	100.0%
	R	Count	13	0	13
		% within County	100.0%	.0%	100.0%
	S	Count	3	0	3
		% within County	100.0%	.0%	100.0%
Total		Count	183	30	213
		% within County	85.9%	14.1%	100.0%
