# Supplementary material for: A multivariate statistical evaluation of actual use of electronic health record systems implementations in Kenya
Source: PLoS One. 2021 Sep 7;16(9):e0256799. doi: 10.1371/journal.pone.0256799 (PMC8423313; doi:10.1371/journal.pone.0256799)
Supplement: Attachment — Submitted filename: Response to Reviewers.docx [file pone.0256799.s008.docx]

 Dear Academic Editor,

**Re: Manuscript PONE-D-21-01911: A multivariate statistical evaluation of actual use of electronic health record systems implementations in Kenya.**

We appreciate the review by PLOS ONE Journal of our Manuscript entitled “A multivariate statistical evaluation of actual use of electronic health record systems implementations in Kenya.” We are grateful for the opportunity to respond comprehensively to the reviewers’ comments. Please find our responses to all the comments by the reviewers below:

**Editor’s Comments**

**1. Please ensure that your manuscript meets PLOS ONE's style requirements, including those for file naming.**

Response: We confirm that we have followed the provided guidelines and conformed to manuscript style requirements.

**2. We note that S1 Appendix in your submission contains map images which may be copyrighted**. **We require you to either (a) present written permission from the copyright holder to publish this figure specifically under the CC BY 4.0 license, or (b) remove the figure from your submission:**

Response: We have uploaded the completed ‘Content Permission Form’ which grants permission to include the map in the manuscript.

**3. Thank you for stating the following in the Acknowledgments Section of your manuscript:**

**'Authors would like to acknowledge KeEMR system developers for providing input into the testing of the study instrument (query script), and helpdesk support to study participants. We also appreciate the support by Kenya Ministry of Health, County health directors, AMPATH Plus and FACES service development partners, and County Health Records Information Officers (CHRIOs). Much appreciation also to all the healthcare facilities and the system champions for their participation in the study.'**

**We note that you have provided funding information that is not currently declared in your Funding Statement. However, funding information should not appear in the Acknowledgments section or other areas of your manuscript. We will only publish funding information present in the Funding Statement section of the online submission form.**

Response: We have removed any funding-related text from the Acknowledgement section.

**4. We note that Figure 1 in your submission contains copyrighted images.**

Response: We have uploaded the completed ‘Content Permission Form’ which grants permissions to include the figure in the manuscript.

**Reviewers’ comments**

**Reviewer #1:**

1. **I read the paper with interest and I can see that there is some interesting data here. However I was not really convinced by some aspects of the work as presented here and found the paper rather too long and a bit repetitious while not really focusing on a clear set of issues and a strong message for the reader.**

Response: Thank you for this feedback. Based on the above comment, we have completely reworked the manuscript in addressing its length as follows:

- Table 2 has been moved to the Appendix as supporting information.
- Revised the Discussion to remove repetitions as shown in the track changes document.

**2. In the introductory section a number of older references are used to set the scene. The paper would be stronger if it focused on more recent studies in its literature review and on literature that serves the needs of this particular study e.g. that details specific metrics of system use, , assessment of system value, and the analysis of log files. Some of what was here felt a bit out-of-date (references over 10 years old).**

Response: We do appreciate reviewer’s comment, and have revised the manuscript to include more recent literature, with almost all referenced publications now being less than 10 years old.

**3. It hardly mentioned core models of system usage and success e.g. DeLone and Mclean get a very brief mention but their model – first and revised version – may be very useful to underpin this work.**

Response: We have taken note of the comments and have provided additional information within the manuscript on the DeLone and McLean Framework. This section has been changed from:

“System use/usage is the utilization of an information system in work processes by individuals, groups or organizations [8,14]. Information System (IS) use is one of the constructs in the revised DeLone &McLean (D&M) IS success model [15]”

to now read:

“Assessment of information system (IS) implementation success is both complex and never a straightforward task [17]. Thus, a range of evaluation methodologies and frameworks have emerged with divergent approaches, strengths, and limitations [18][19]. DeLone & McLean (D&M) IS success model is a mature and validated model for measuring health information systems success that was established in 1992 and revised in 2003 [20]. The model has been used to evaluate implementation success for a wide range of health information systems. Berhe *et al.,* recently used the model to evaluate EMRs effectiveness from a user’s perspective in Ayder Referral hospital in Ethiopia [21]. Cho *et al*. also used the model to evaluate the performance of newly-developed information systems in three public hospitals in Korea [22].

The revised D&M model has six dimensions used to measure IS implementation success, namely: *System quality, Information quality, Service quality, System Use/intention to use, User satisfaction* and *Net benefits*. Of these dimensions, *‘System Use*’ was identified as the most appropriate variable for measuring the success of IS [20][23]. *System use* is the utilization of an IS in work processes by individuals, groups or organizations [11].” (Page 4, Lines 76-90)

4. **I would be more interested in the data that is collected here if it was not described (on a couple of occasions) as ‘objective’ or ‘un biased’. From my position this data is useful, interesting, but it is not a higher truth than other data collected in other ways. Nor is it (or any other data) un-biased – computer systems and their data are biased from the day they are developed!** **I would like more discussion of the validity and potential of the data and independent variables, and how they might be interpreted or add value. But this cannot be asserted or taken for granted..**

Response: We apologize for any confusion caused by these descriptions. In this manuscript, we meant to emphasize that the indicators were generated purely using data alreaddy recorded within the EHRs. In response to the reviewer’s comments, we have removed all references to the data being objective or unbiased.

Validity of the data is discussed in the methods section from line 170 (page 8) and Data collection and analysis section lines 188-190 (page 9).

**5. Similarly I felt that the focus on the GLM model was the weakest part of the paper. To reduce the complex reality of clinical work and use of EHR over years to a single model seems a rush to judgement. The discussion the Table 4’s findings seemed to be more description than an evaluation or analysis of the results (although the discussion section did take this a little further – e.g. page 17). Before any such model, I would suggest more attention paid to the data itself and to the various correlations that it reveals**.

Response: We have taken note of the comments on GLM and we apologize for the confusion. The main purpose of the analysis was to get insights as to whether EHRs use was influenced by the characteristics of the facilities under study. To capture this kind of relationship, we found GLM to be a more suitable model due to the variables under evaluation containing both continuous and categorical data. Moreover, GLM also reveals relationship in the data and in our study, it shows how system usage can be predicted. Training on additional data will help fine tune the model.

**6. I was not clear what the section on ‘Implementation by county (page 11 and 12) added to the paper – just a large table . The data on implementation year is interesting but could find a home elsewhere in the paper.**

Response: The reviewer’s comments are noted. In reporting the study findings, we aimed to go beyond simple descriptive reporting of indicator performance to delineating factors that affected the usage performance for the various indicators. We agree with the reviewer on the comment regarding the large table (‘*Implementation by County’*), and we have moved the table to the Appendix (S3-Appendix) that provides a reference to the distribution of the various implementations.

7. **I was puzzled by the definition of some variables. For example, is the ‘EHR Variable completeness’ (page 13) really 100%in any meaningful way? Is the fact that then data fields are defined in the software really enough to say this? A real measure might be the number of these fields that are actually in use – e.g. populated in over x% of patient records?**

Response: We appreciate the reviewer’s comments on ‘EHRs Variable Completeness’ indicator. We do agree measuring the number of the data fields that are in actual use could be another measure, but want to also point out that many of the fields within the EHRs are optional and having these empty does not mean that the data is incomplete. The ‘EHR Variable Completeness’ indicator as defined in this paper conforms to the country-specified definition for the 23 data elements that are required for all HIV positive patients and are used for reporting at the national level. This is outlined in Table 1 of the manuscript under ‘Indicator Measure’ that describes ‘EHR Variable Completeness’ as ‘The extent to which all required data elements for a patient are contained within the EHRs.’ This indicator is derived in line with the description of the indicators as outlined in *Ngugi et al.* [*https://doi.org/10.1371/journal.pone.0244917*](https://doi.org/10.1371/journal.pone.0244917)*).*

**8. I was also puzzled by the Observations measures (page 13). I would have thought this would need a denominator to of any interest – and what the denominator should be would be (patient encounters, clinical staff-users etc) is an interesting issue to address in the paper at some depth, but it only appears briefly in the discussion (page 17).**

Response: We appreciate reviewer’s comments on Observations indicator. We want to highlight that in this manuscript, we are not defining or revising indicators, but are using the approved usage indicators as defined in *Ngugi et al.* [*https://doi.org/10.1371/journal.pone.0244917*](https://doi.org/10.1371/journal.pone.0244917)*).* In the *Ngugi et al* paper and summarized in Table 1, the Observations indicator is defined as ‘Number of mandatory HIV-related clinical data elements recorded for patients in the EHRs during the reporting period’. These definitions and indicators were reached after an extensive and consultative process described in the Ngugi *et al* paper. To avoid confusion in meaning, we have added in brackets the term ‘Clinical data volume’ to this Indicator for clarity. In response to the reviewer’s comments, we have included the factors that affect clinical data in the discussion:

“Several factors determine the volume of electronic data from each facility such as patients’ volume, frequency of patients’ visits (encounters), EHRs mode of use, and active usage of the system during care.” (page 17, lines 363-365).

**9. In summary I believe that the paper could be significantly improved by tightening the presentation and some of the repetition of findings, focusing on interpreting the data more and less on the GLM. I would also suggest a stronger discussion section that takes the reader further and focuses on the ‘so-what ‘. The present version seems to repeat the main findings, but not take the argument further.**

Response: We take note of the comments by the reviewer. As per earlier comments by the reviewer, we have addressed repetitions as outlined in Response #1 above and addressed GLM comments as outlined in responses to comment #5. In addition, we have extensively edited the Discussion to address the ‘so-what’ factor and to reduce any repetitions. We highlight our edits in the Discussion using track changes. We hope that you find these changes satisfactory.

**Reviewer #2**:

**Thank you for the opportunity to review this manuscript on actual use of electronic health records systems. Since the advent of electronic health records, there have been concerns about the benefits of using EHRs and the success of implementation of EHRs. In the research conducted, the authors examined seven indicators that might reflect the actual use of electronic health records.**

1. **Upon reading the manuscript, the question arises whether the authors are really identifying the actual use of EHRs by health care staff with the seven indicators they have chosen.**

Response: We thank the reviewer for this comment. This evaluation is not limited to the use of EHRs by health care staff (as they are just one group that utilizes the system). The study evaluates multiple aspects of actual system use, which also include other users and additional EHRs use-cases, such as reporting and information exchange. The study employs a vetted set of indicators that were determined consultatively with a broad range of stakeholders (Ngugi *et al*, 2021, <https://doi.org/10.1371/journal.pone.0244917>).

2. **The first indicator is ‘staff system use’ and was measured by the percentage of facility staff members who used the EHRs during the reporting period. In their study, the authors found a low average of 18.2%. However, this number has little meaning without providing more information about the context. For instance, are the health care facilities using a few stationary computers or are they using a shared or individual iPad? With a stationary computer health care professionals often log in with one user account and subsequently use the EHRs with multiple professionals on one user account. This is also the case when working with a shared iPad. Therefore, the ‘staff system use’ as measured by the authors is not an accurate indicator for actual use of the EHRs.**

Response: We have taken note of the comment and agree that there would have been cases where staff share devices. However, the log-in credential we are looking at is actual log-in into the EHRs (for which individuals have their own passwords) – this is not the same as the shared password sometimes used to log into a single computer or device. As such, the low average measure of providers logging into the EHRs using assigned individual passwords. is of concern. In agreement with the reviewer’s comment, it is possible that providers could be sharing individual-level passwords to log into the EHRs, and this would contribute to the low ‘Staff system use’. Such usage of passwords would be highly problematic and would need to be addressed. We have revised the paragraph to incorporate this and recommended training on clearing dormant user accounts and the importance of not sharing login credentials. We have also recommended further research to establish user-computer ratio. The manuscript now reads:

“The finding warrants deeper assessment on user credentialing processes and account usage patterns (such as sharing of credentials). It also highlights the need for training users on appropriate use of account credentials and of system administrators on mechanisms to monitor and manage the various user accounts and system access. We also recommend further research to establish user-computer ratio in the care facilities”. (Page 16/17, lines 348-352)

3. **In addition, another indicator is ‘standardized terminologies’. This indicator is measured through the proportion of key terminologies that are mapped to standard terminologies. Mapping of terminologies is mostly relevant for the exchange of health care information and for the use of routine care data for research purposes. However, this mapping has no direct influence on the use of EHRs by health care staff in daily care practice. So, how does this indicator say anything about the actual use of EHRs? That remains unclear in this study. Moreover, there are different standardized terminologies for doctors, nursing staff and other health care staff. The authors lack to explain by which health care staff the EHRs are being used. Therefore, the indicator standardized terminology remains vague and unclear.**

Response: This comment is similar to comment #1 by this reviewer, and we apologize for the confusion. We highlight once again that EHRs usage evaluation is not limited to system use just by health care workers, but also touches on other uses of EHRs such as for health information exchange. We hope this clarifies the use of this indicator, which is also one that is part of the developed indicator set (Ngugi *et al* 2021, <https://doi.org/10.1371/journal.pone.0244917>).

4. **The authors already mentioned themselves that the study findings are not generalizable to other countries. They suggest that their approach for analysing EHRs use can be generalizable to other countries. However as explained before, this approach seems very questionable. Therefore, the added value of this manuscript for the international research community is not clear.**

Response: We take note of the reviewer’s comment and have made extra efforts within the manuscript to highlight why this work is relevant. In the manuscript we now state:

“In most LMICs, measure of success of EHRs scale-up often relies on simple counts of the number of EHRs implementations. This study demonstrates that: (a) through use of standardized indicators,[26] key new insights and gaps on actual status of EHRs implementations within countries use can be identified; (b) aspects of national-level EHRs usage assessments need not be time- or resource-intensive, as assessments can be automated using data already within the EHRs; and (c) mechanisms that allow efficient EHRs usage assessments offer insights to enable any identified EHRs usage gaps to be addressed in a timely manner.”(Page 5, lines 98-105)

We appreciate the comment on generalizability and agree with the reviewer that it was confusing. We have revised the limitations section to be clearer as outlined below:

“The study does, however, acknowledge a few limitations. It was only conducted in one country (Kenya) and the findings do not necessarily translate directly to other countries. However, the study provides demonstration case that can be modelled by other countries to inform similar EHRs usage evaluations.” (Page 18, lines 383-387)

5. **Use of abbreviations. The authors are not consistent in use of abbreviations for the electronic health records. They use both EHR, EHRs and EMRs which causes confusion. Besides, the authors use a lot of other abbreviations which is not beneficial for the readability of the manuscript. The authors should only use universally used abbreviations and only when necessary. This applies both to the body of text and the tables.**

Response: We apologize for the inconsistency, and have settled on using EHRs in the entire manuscript. We have also written in full other abbreviations that may cause confusion to readers. All other abbreviations used in the manuscript are now also defined in full when first mentioned.

6. **In the ‘Introduction’ section the authors write that EHRs improve quality of care and support HIV programs at a national level. Authors should explain these suggested relations further, since it remains unclear.**

Response: We appreciate the reviewer’s comments and have provided additional information to support the statements that EHRs improve quality of care. These additions are highlighted below:

“For instance, introduction of Electronic Medical records systems (EMRs) in health care has shown improvement in time dependent events such as patient waiting time, time to processing specimen in the laboratory from test request to results reporting among others benefits [7], [8]. Moreover, a systematic review on utilization of EHRs for public health in Asia revealed the ability to help identify and predict seasonal outbreaks and high risk areas and prevent infections or diseases, leading to better health outcomes [9]

”. (page 3 lines 50-55).

We have also added additional information on HIV program support by EHRs. The manuscript now states:

"However, adoption of EHRs in Sub-Saharan Africa are largely driven by HIV treatment international programs such as President’s Emergency Plan for AIDS Relief (PEPFAR) to support patient data management [11][12].” (page 3, lines 59-62)

7. **In the ‘Introduction’ section the IS success model is introduced without further describing the model. For readers who are not familiar with this model this paragraph is unclear. Further explanation is needed.**

Response: We appreciate and have taken note of the comments. We have added further description as guided and updated the manuscript as follows:

“Assessment of information system (IS) implementation success is both complex and never a straightforward task [17]. Thus, a range of evaluation methodologies and frameworks have emerged with divergent approaches, strengths, and limitations [18][19]. DeLone & McLean (D&M) IS success model is a mature and validated model for measuring health information systems success that was established in 1992 and revised in 2003 [20]. The model has been used to evaluate implementation success for a wide range of health information systems. Berhe *et al.,* recently used the model to evaluate EMRs effectiveness from a user’s perspective in Ayder Referral hospital in Ethiopia [21]. Cho *et al*. also used the model to evaluate the performance of newly-developed information systems in three public hospitals in Korea [22].

The revised D&M model has six dimensions used to measure IS implementation success, namely: *System quality, Information quality, Service quality, System Use/intention to use, User satisfaction* and *Net benefits*.” (page 4 lines 76-88).

8. **In the ‘Material and Methods’ section the authors write there are two types of EHRs endorsed for national development. It is unclear what was meant by ‘two types’. Does this mean EHRs from two different vendors?**

Response: Per the reviewer’s comment, we have added a clarification on this as follows:

“While two EHRs (KenyaEMR and IQCare) by different vendors were initially endorsed for national deployment in support of HIV care, the country has since 2019 transitioned to supporting and deploying only KenyaEMR system (KeEMRs).” (Page 5, line 114)

We truly appreciate the feedback from the reviewers which have strengthened our manuscript. We hope that you will find our responses satisfactory. Thank you once again for considering our manuscript in PLOS One.

Sincerely,

Philomena Ngugi

waruharip@gmail.com

Corresponding author
